# Rapid and Simple Detection of *Mycobacterium avium* subsp. *paratuberculosis* Using a Lateral Flow Assay Based on CRISPR-Cas12a Combined with Recombinase Polymerase Amplification or Nested PCR

**DOI:** 10.3390/pathogens15010024

**Published:** 2025-12-24

**Authors:** Yue-Rong Lv, Yi-Yang Liu, Rong Zhang, Bo Yang, Shi-Yuan Xue, Yu-Lin Ding, Jun-Tao Jia, Hasi Bayaer, Alateng Bagen, Rui-Bin Chen, Siqin Tunala, Li Zhao, Yong-Hong Liu

**Affiliations:** 1 College of Veterinary Medicine, Inner Mongolia Agricultural University, Hohhot 010018, China; lyr765619420@163.com (Y.-R.L.); 18702243475@163.com (Y.-Y.L.); xsy55145600@163.com (S.-Y.X.); dingyulin2001@126.com (Y.-L.D.); juntaojia1982@imau.edu.cn (J.-T.J.); 2 Otok Banner Animal Disease Prevention and Control Center, Ordos 016100, China; zr_129@163.com (R.Z.); 13947741701@139.com (H.B.); 13734774882@139.com (A.B.); cyb_0921@163.com (R.-B.C.); nmhfei@126.com (S.T.); 3 Animal Disease Control Center of Ordos, Ordos 016100, China; 15886840959@126.com

**Keywords:** *Mycobacterium avium* subsp. *paratuberculosis*, CRISPR-Cas12a, RPA, nested PCR

## Abstract

Paratuberculosis (PTB), caused by Mycobacterium avium subsp. paratuberculosis (MAP), is a chronic intestinal disease in ruminants. PTB is difficult to diagnose, control, and eradicate, leading to substantial economic losses. Thus, sensitive and specific detection methods are urgently required. crRNA and primers targeting the MAP ATPase FtsK gene were designed for recombinase polymerase amplification (RPA) and nested PCR. Fecal DNA was amplified using RPA or nested PCR, purified with Tris-saturated phenol-chloroform-isoamyl alcohol, and detected via CRISPR-Cas12a. Moreover, signals were read using a qPCR instrument, fluorescence reader, or lateral flow strips. RPA–CRISPR-Cas12a and nested PCR–CRISPR-Cas12a assays were optimized and validated on 50 clinical samples and 7 MAP cultures. The limits of detection were 1 × 10^−10^ μg/μL for RPA–CRISPR-Cas12a and 1 × 10^−14^ μg/μL for nested PCR–CRISPR-Cas12a. Efficient cleavage of the ssDNA reporter occurred at DNA concentrations of ≥1 × 10^−4^ μg/μL, producing a strong fluorescent signal. All three detection methods showed perfect agreement with reference assays across both sample sets. This study presents the first integration of RPA or nested PCR with CRISPR-Cas12a for MAP detection, enabling rapid, specific, and highly sensitive diagnosis. Flexible detection options allow adaptation to available resources and bacterial loads, supporting practical use in PTB control.

## 1. Introduction

Johne’s disease or paratuberculosis (PTB), is chronic granulomatous enteritis with mesenteric lymphadenitis, mainly caused by *Mycobacterium avium* subsp. *paratuberculosis* (MAP) in wild and domestic ruminants. Infected animals have persistent diarrhea and exhibit progressive weight loss, eventually succumbing to the disease [1,2]. These symptoms not only impair livestock productivity and compromise immune function [3,4], but also pose a risk to human health through the transmission via animal-derived products [5]. PTB is often under-recognized in ruminants and is a hidden threat to small ruminants [6], as well as a zoonotic disease with major effects on animal welfare, economic productivity, and public health [1,7].

PTB develops through the following four stages: silent infection, subclinical disease, clinical stage, and advanced clinical stage [8]. During the first two stages, MAP bacteria are shed intermittently at low bacterial levels [7]. Thus, detecting subclinical PTB is critical for disease control [9]. Current MAP detection methods include microbial culture, acid-fast staining with microscopy, serological assays [10], conventional PCR, and quantitative real-time PCR (qPCR) [7]. MAP confirmation by culture, the gold standard, requires 3–4 months [11]. Moreover, immunological tests are limited to identifying animals during the subclinical disease stage owing to the lack of measurable antibody titers against MAP [12].

In 2012, genome-editing tools based on clustered regularly interspaced short palindromic repeats (CRISPR) and CRISPR-associated proteins (Cas) were introduced [13]. With its programmability and precise genomic targeting, CRISPR rapidly became a powerful tool for molecular diagnostics [14]. Cas12a binds crRNA and activates transnuclease shearing activity [15]. This activity nonspecifically cleaves single-stranded DNA reporters, including fluorescent probes and biosensors, thus amplifying detection signals [16,17]. The CRISPR-Cas12a system allows specific recognition of DNA sequences with simultaneous signal amplification, enabling highly sensitive and specific pathogen detection [18].

**Figure 1:**
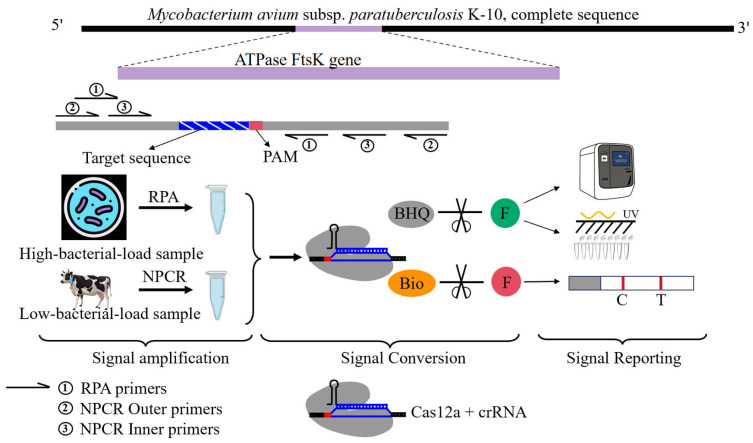
Simplified schematic of the technical workflow of this study.

In recent years, isothermal amplification has been integrated with CRISPR-Cas12a to develop the DNA endonuclease-targeted CRISPR trans reporter system, i.e., DETECTR, for rapid, highly sensitive pathogen detection [19]. A more convenient one-step assay linking RPA and CRISPR-Cas12a has also been developed [20], where Cas12a is encapsulated in a gel matrix for integrated single-step detection [21]. Results can be visualized through lateral flow assays, but the assay’s performance depends heavily on RPA efficiency [22]. This study aims to establish a flexible, convenient, and efficient MAP detection system by integrating RPA or nested PCR with CRISPR-Cas12a to achieve two-step target DNA signal amplification, followed by visualization with lateral flow assay, allowing detection across varied sample types and conditions (Figure 1).

## 2. Materials and Methods

### 2.1. DNA Extraction

Genomic DNA was extracted from pretreated fecal samples in a biosafety cabinet using the E.Z.N.A.^®^ Stool DNA Kit (Omega BioTek Inc., Norcross, GA, USA) following the manufacturer’s protocol and stored at −20 °C for later use. Moreover, tissue samples were processed for DNA extraction using the TaKaRa MiniBEST Universal Genomic DNA Extraction Kit Ver. 5.0 (TaKaRa, Beijing, China). Bacterial colonies were subjected to DNA extraction using the MiniBEST Bacteria Genomic DNA Extraction Kit (TaKaRa).

### 2.2. Design and Preparation of crRNA and Primers

**Figure 2:**
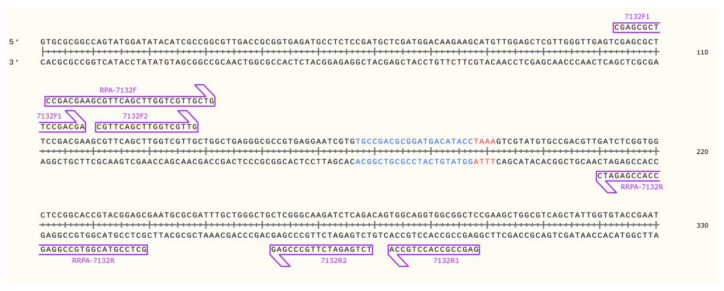
Positional relationship of each primer relative to the target sequence. Target sequence and PAM site are shown in blue and red, respectively.

*ATPase FtsK* gene [23] was selected as the target sequence for crRNA design. A 20-nucleotide sequence located downstream of a T-rich protospacer adjacent motif (PAM) was chosen as the target site, and a corresponding hairpin structure was designed according to the specific Cas12a enzyme used. Primers for RPA and nested PCR were designed based on the target region using Primer Premier 6. Three primer pairs were designated as follows: RPA-7132F (CCG ACG AAG CGT TCA GCT TGG TCG TTG CTG)/RPA-7132R (GCT CCG TAC GGT GCC GGA GCC ACC GAG ATC); 7132F1 (CGA GCG CTT CCG ACG A)/7132R1 (GAG CCG CCA CCT GCC A); and 7132F2 (CGT TCA GCT TGG TCG TTG)/7132R2 (TCT GAG ATC TTG CCC GAG) (Figure 2). To verify in silico that the two chosen genomic regions were unique to MAP, the target sequences were analyzed using the Basic Local Alignment Search Tool (BLAST) program (https://blast.ncbi.nlm.nih.gov/Blast.cgi, accessed on 12 July 2025) and aligned against the GenBank (http://www.ncbi.nlm.nih.gov, accessed on 12 July 2025) nucleotide database to confirm specificity.

### 2.3. RPA Assay and Nested PCR

RPA method was used for DNA extracted from samples with high bacterial loads, such as bacterial colonies, to enrich target dsDNA under isothermal conditions. The TwistAmp^®^ (TwistDx, Hertfordshire, UK) Basic Kit was used according to the manufacturer’s instructions. The 50 μL reaction volume contained 29.5 μL of rehydration buffer, 2.4 μL of 10 μM of each primer (RPA-7132F/RPA-7132R), 1 μL of extracted DNA, 2.5 μL of 280 nM magnesium acetate (MgOAc), and sterile nuclease-free water up to 50 μL. The reaction was run at 37–42 °C (six temperature points at 1 °C intervals) for 20 min. MgOAc was added to the inner tube lid, whereas the other reagents were placed at the bottom of the tube before capping. A brief spin was used to mix MgOAc with the solution, initiating the RPA reaction.

When RPA products did not effectively activate LbCas12a transcleavage activity or when DNA was extracted from samples suspected to have low bacterial load, the RPA amplification system was replaced with nested PCR. The 25 μL reaction volume contained 12.5 μL of Takara Premix Taq™ (TaKaRa, Beijing, China), 9.5 μL of ddH_2_O, 1 μL of 10 μM forward and reverse primers, and 1 μL of DNA template. For preamplification, primers 7132F1 and 7132R1 were used under the following conditions: initial denaturation at 94 °C for 2 min; 25 cycles of 94 °C for 30 s, annealing from 57.6 °C to 62.4 °C (in 0.4 °C increments for optimization) for 30 s, and extension at 72 °C for 30 s; followed by final extension at 72 °C for 3 min. For nested amplification, primers 7132F2 and 7132R2 were used under the same conditions, except that annealing was performed from 52.6 °C to 57.4 °C in 0.4 °C increments. The target fragment was successfully amplified. Amplicons from both methods were extracted with an equal volume of Tris-saturated phenol-chloroform-isoamyl alcohol (25:24:1) and centrifuged at 12,000 rpm for 5 min. The resulting supernatant was used as target DNA in the LbCas12a system.

### 2.4. LbCas12a/crRNA Transcleavage Assay

Two detection systems—machine-based detection and lateral flow test strip detection—were established based on the DNA reporter modifications. In the machine-based detection system, a 30 μL reaction volume was prepared according to the manufacturer’s instructions for LbCas12a (Cpf1; EDITGENE, Guangzhou, China): 1 μL of 1 μM LbCas12a nuclease, 1 μL of 1 μM purified crRNA, 1 μL of target DNA (unpurified RPA or nested PCR products), 3 μL of 4 μM DNA reporter (FAM-TTTTTTTTTTTT-BHQ1), 3 μL of 10× cleavage buffer, and DEPC-treated H_2_O up to 30 μL. Reactions were run in a qPCR instrument at 37 °C, recording fluorescence every 30 s for 30 min. Alternatively, reaction tubes were incubated at 37 °C for 20 min in a constant-temperature device and inspected for fluorescence under ultraviolet (UV) light.

The test strip detection system was established based on the manufacturer’s instructions for the CRISPR Single-Target Nucleic Acid Test (Strip Tolo Biotech, Shanghai, China). The 50 μL volume contained 1 μL of 10 μM LbCas12a nuclease, 1 μL of 10 μM purified crRNA, 1 μL of target DNA (unpurified RPA or nested PCR products), 1–5 μL of 10× cleavage buffer (optimized at 1 μL), 1–20 μM DNA reporter (FAM-TTTTTTTTTTTT-biotin; recommended concentration: 5 μM), and DEPC-treated H_2_O to 50 μL. After incubation at 37 °C for 20 min in a constant-temperature device, test strips were inserted, allowed to develop for 5 min, and visually assessed for control and test lines.

To test sensitivity, standard target DNA fragments for RPA and nested PCR were synthesized by a commercial provider (Sangon Biotech, Shanghai, China). Purified fragments were serially diluted and amplified using RPA and nested PCR, with the resulting amplicons introduced into the CRISPR-Cas12a detection system and analyzed via fluorescence signal measurement and lateral flow test strip reading. This process enabled determination of the detection limit for both systems.

### 2.5. Clinical Sample Validation

To assess the developed method under complex conditions, RPA–CRISPR-Cas12a and nested PCR–CRISPR-Cas12a assays were applied to detect MAP in sheep fecal samples and bacterial colony isolates. In total, 50 fecal samples previously validated via qPCR were randomly selected, including 22 positive and 28 negative samples [7]. Seven MAP colony isolates from solid media cultures were confirmed in our laboratory. All samples were tested via RPA–CRISPR-Cas12a and nested PCR–CRISPR-Cas12a, and results were compared with qPCR outcomes. Additionally, nested PCR amplicons were Sanger sequenced by a commercial provider (Sangon Biotech, Shanghai, China), and sequence identities were confirmed using BLAST (Version 2.16.0).

## 3. Results

### 3.1. Optimization of Reaction Conditions

The optimal amplification efficiency for the RPA reaction was achieved at 39 °C. For nested PCR, optimal annealing temperatures were 60 °C for preamplification and 55 °C for nested amplification. In the lateral flow test strip assay, the most stable signal was obtained with a probe concentration of 10 μM and 4 μL of 10× cleavage buffer in the reaction system.

### 3.2. Sensitivity and Specificity of the RPA– or Nested PCR–CRISPR-Cas12a Detection Platforms

**Figure 3:**
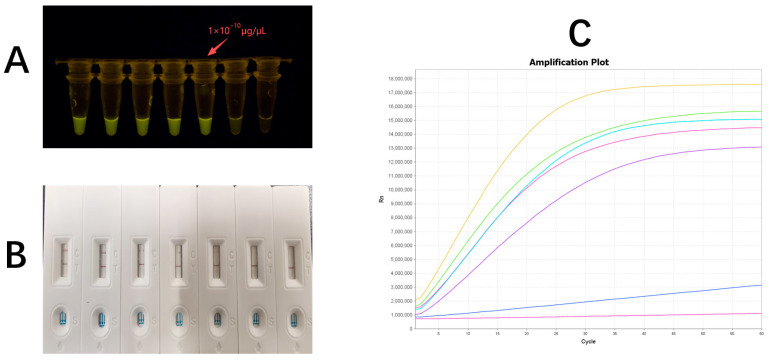
Determination of the limit of detection. (**A**) Fluorescence observation method, (**B**) test strip detection method, and (**C**) instrument-based detection method.

RPA–CRISPR-Cas12a platform had a sensitivity of ≤10^−10^ μg/μL for the target gene, whereas the nested PCR–CRISPR-Cas12a platform achieved a higher sensitivity of ≤10^−14^ μg/μL. The three signal readout methods (Figure 3) showed differences in sensitivity, and compared with the lateral flow test strip method, instrumental detection and fluorescence observation were each one order of magnitude more sensitive. When DNA concentration in the CRISPR-Cas12a reaction system was ≥1 × 10^−4^ μg/μL, the ssDNA reporter probe was efficiently cleaved, producing a detectable fluorescence signal.

### 3.3. Clinical Performance Evaluation of MAP RPA– or Nested PCR–CRISPR-Cas12a Detection

**Figure 4:**
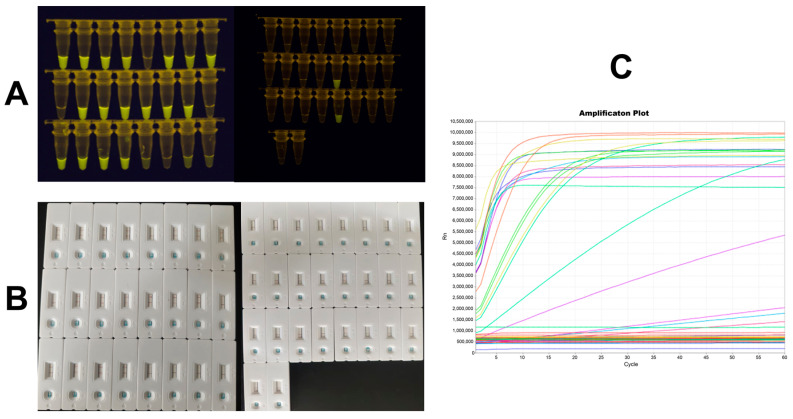
Detection results from fecal sample analysis. (**A**) Fluorescence observation method, (**B**) test strip detection method, and (**C**) instrument-based detection method.

**Figure 5:**
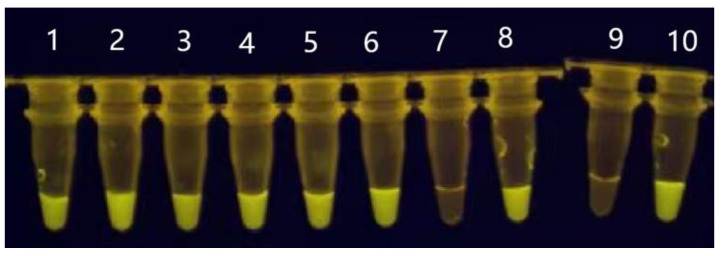
Detection results from colony sample analysis. Tubes 1–6 and 8: DNA from bacterial colonies; tube 7: DNA from MAP-positive fecal sample; tube 9: negative control; tube 10: positive control.

All three readout methods of the nested PCR–CRISPR-Cas12a assay yielded identical results, exhibiting 100% concordance with qPCR (Figure 4). The RPA–CRISPR-Cas12a assay detected all seven MAP colonies with positivity (Figure 5), achieving 100% agreement with qPCR when applied to colony samples. Sequence analysis confirmed that all 22 positive samples were positive for MAP.

## 4. Discussion

PTB was first described in the 19th century [24], and its causative agent was formally identified as MAP in 1923 [25]. PTB has a worldwide distribution [26]; it primarily affects ruminants but has been reported in rabbits, foxes, stoats and weasels [27]. During the prolonged PTB incubation period, infected animals may remain asymptomatic for 2–7 years [28], yet actively shed MAP in milk and feces [29]. Furthermore owing to its heat-, cold-, and desiccation-resistant traits, MAP persists in soil for >1 year and may survive longer in aquatic environments [30], causing sustained environmental contamination [1]. These factors have contributed to a “don’t test, don’t tell” practice in the industry, leading to increased PTB prevalence [31]. Even subclinical infection markedly reduces animal performance and results in extensive direct and indirect economic losses in the breeding industry [3]. For example, subclinical infection can reduce milk yield and quality, lower slaughter value, impair fertility, and weaken immune resistance [4]. In the USA, annual economic loss per infected cow is reportedly 21–78 US dollars, whereas in certain European countries, such as France, such losses can reach 251 US dollars [32]. Moreover, evidence indicates that MAP is associated with a growing spectrum of human diseases, including Crohn’s disease, sarcoidosis, Blau syndrome, autoimmune diabetes, autoimmune thyroiditis, systemic lupus erythematosus, multiple sclerosis, rheumatoid arthritis, and Parkinson’s disease [33]. Thus, it is necessary to detect MAP as early as possible so that effective measures can be taken earlier to reduce economic losses and enhance the protection of human and animal health.

Among current diagnostic methods, culture remains the gold standard for PTB confirmation; however, it requires 3–4 months to yield results [11,34], substantially delaying timely disease control. Available immunodiagnostics primarily rely on protein antigens from the *M. avium* complex [35], which exhibit limited specificity. Molecular methods provide higher specificity and sensitivity compared with culture or immunodiagnostics [11]. Conventional PCR assays were initially designed to target the IS900 insertion element; however, IS900-like sequences in other mycobacterial species [36,37] compromise specificity unless primers are carefully designed [20]. SYBR Green qPCR has been applied to directly detect MAP in fecal samples [38]; however, specificity is primer-dependent and limited by nonspecific amplification [39]. Probe-based qPCR offers high sensitivity and specificity [40], with a LOD for MAP as low as 0.0002 fg/μL, and completes the entire detection process within 1.5 h [23]. However, cost per sample can reach 5.84 USD, and the method requires specialized instrumentation, making it impractical for large-scale testing and inaccessible to resource-limited laboratories.

The RPA/nested PCR-CRISPR-Cas12a addresses the aforementioned limitations. By incorporating a two-step amplification strategy, LOD in our method was one order of magnitude lower than that of probe-based qPCR, yet remains substantially higher than that of conventional PCR assays. Using Cas12a transcleavage activity ensures that only target sequences activate the enzyme, achieving “no signal without target” and enhancing specificity. The reaction is conducted at a constant temperature of 37–39 °C, and results can be visualized using lateral flow test strips or through UV light irradiation, reducing reliance on sophisticated laboratory equipment. Compared with agarose gel electrophoresis or sequencing of amplification products, our method streamlines the workflow and shortens the time (3.5 h) required for sample analysis. Compared to probe-based methods, although the assay time is extended by 2 h, the cost per sample is reduced to 0.8 USD.

Furthermore, Cas12a’s transnuclease activity has been widely used for sequence-specific nucleic acid detection, enabling sensitive and programmable diagnostics [41]. The present study shows that when template DNA in the CRISPR-Cas12a system is ≥1 × 10^−4^ μg/μL, the ssDNA reporter is efficiently cleaved, producing a detectable fluorescence signal. Below this threshold, Cas12a transnuclease activity is insufficient, leading to weak or undetectable signal output. Therefore, preamplification of target DNA is required. RPA is commonly integrated with CRISPR-Cas12a to streamline pathogen detection and has demonstrated high efficacy in detecting viruses [42], parasites [43], and certain bacteria [44], with reported LODs as low as 10^−1^ copies/μL in some studies [45]. In the current study, the RPA–CRISPR-Cas12a method showed limited sensitivity, with a LOD of 1 × 10^−10^ μg/μL. It produced strong amplification with bacterial colonies but was less effective when applied to complex fecal DNA. Different DNA targets, even those with identical GC content, primer melting temperatures, and amplicon lengths, can exhibit highly variable amplification efficiencies, with the underlying mechanisms not fully understood [46]. Although RPA–CRISPR-Cas12a is less effective for direct detection in fecal samples, it is suitable for high-bacteria-load specimens. To address the challenges posed by complex matrices and low pathogen abundance, the CRISPR-Cas12a detection step was combined with nested PCR preamplification. Serial dilution experiments established a LOD of 1 × 10^−14^ μg/μL for the nested PCR–CRISPR-Cas12a assay. Although this approach extends the assay time compared with RPA-based methods, it greatly enhances sensitivity and enables detection of low-abundance MAP in complex samples with minimal instrumentation requirements. Visual readout via lateral flow test strips achieved a lower LOD than that obtained via instrumental fluorescence. However, in resource-limited settings, UV-based fluorescence observation still enables reliable interpretation, providing flexibility across laboratories.

## 5. Conclusions

To the best of our knowledge, this study is the first to report a method integrating RPA or nested PCR with CRISPR-Cas12a technology for MAP detection. For high-bacterial-load samples, such as bacterial colonies, the RPA–CRISPR-Cas12a method was used, reducing the entire detection process to ≤1 h. High detection specificity was achieved through the sequence-specific binding of crRNA, target DNA, and the Cas12a complex. For low bacterial-load samples, such as feces, the nested PCR–CRISPR-Cas12a method was applied, eliminating the need for traditional procedures, e.g., nucleic acid gel electrophoresis and sequencing, while achieving high specificity and sensitivity. The developed method enabled result visualization through three approaches, namely instrumental detection, fluorescence observation, and lateral flow test strip detection, providing an effective and practical tool for precise early diagnosis of PTB.

## Data Availability

The data presented in this study are available on request from the corresponding author.

## Abbreviations

PTB: Paratuberculosis; MAP: *Mycobacterium avium* subsp. *paratuberculosis*; PCR: polymerase chain reaction; RPA: recombinase polymerase amplification.
